# Successful Radiofrequency Ablation of the Right Lateral Accessory Pathway with Modified Carpentier Reconstruction Surgery in a Patient with Ebstein Anomaly Concomitant of Secundum Atrial Septal Defect, Atrial Fibrillation, and Wolff–Parkinson–White Syndrome

**DOI:** 10.1155/2022/8343943

**Published:** 2022-04-04

**Authors:** Van Dan Nguyen, Xuan Tuan Nguyen, Van Tung Pham, Le Tra Pham

**Affiliations:** Hanoi Heart Hospital, Hanoi Medical University, Vietnam

## Abstract

Ebstein anomaly (EA) results from the failure of proper delamination of the tricuspid valve leaflets from the right ventricle (RV) myocardium. The severity of EA occurs on a spectrum that results in varying degrees of tricuspid regurgitation, atrial dilation, RV dilation, and dysfunction. These effects have the potential to create substrates that can give rise to atrial arrhythmia, ventricular arrhythmia, and a greater incidence of Wolff–Parkinson–White (WPW) syndrome Wackel et al. (2018) accounting for 0.5% of all congenital heart diseases (Oh et al. 1985). In the case of atrial fibrillation and WPW, it is very dangerous for the patient because of hemodynamic compromise, syncope, and sudden death. In this case report, we share our experience in using radiofrequency ablation to ablate right lateral accessory pathway, with modified Carpentier technique in operation to treat an adult patient diagnosed with Ebstein anomaly, atrial septal defect, atrial fibrillation, and WPW syndrome.

## 1. Case Report

**Figure 1:**
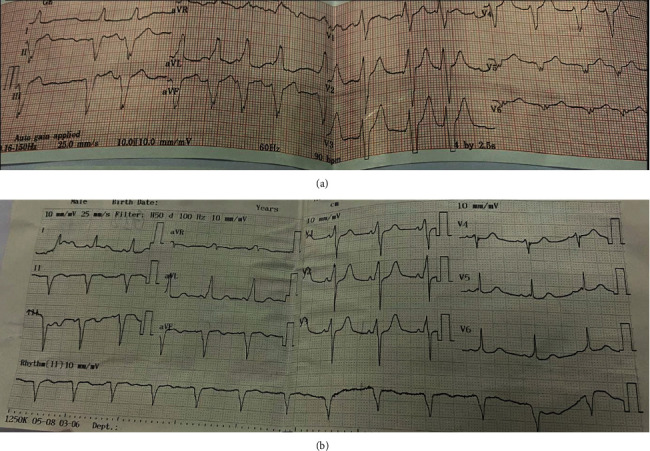
(a) ECG shows irregularly wide QRS tachycardia with ventricular rate ranging from 130 to 150 bpm and delta wave. (b) ECG shows a sinus rhythm heart rate of 70 bpm, delta waves, wider QRS, and short PR suggesting WPW syndrome with the right side accessory pathway.

**Figure 2:**
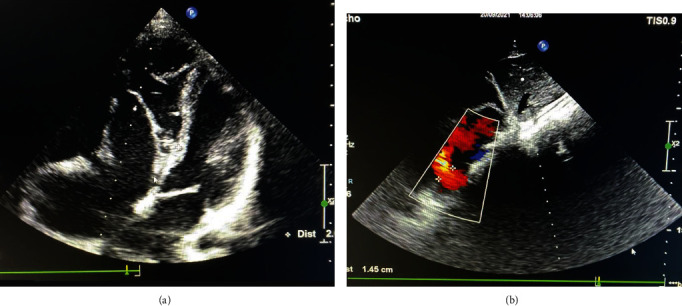
(a) Echocardiogram shows situs solitus, levocardia, left side aortic, and Ebstein types B-C. (b) ASD size of 8.3 mm with left to right shunt.

A 19-year-old male patient was admitted to our emergency department for palpitation, shortness of breath, presyncope, and history of 3 episodes of syncope. The following are his vital signs: irregular heart rate of 130-150 bpm, blood pressure of 70/50 mmHg, and respiratory rate of 25 bpm. Emergency POCUS shows the following: stunning left ventricular, Ejection Fraction (EF) of 20%, no obstruction left ventricular outflow tract, Ebstein type B, and right to left shunt atrial septum defect (ASD). Electrogram (ECG) shows irregularly wide QRS tachycardia with ventricular rate ranging from 130 to 150 bpm and delta wave (Figure 1(a)). So we diagnosed atrial fibrillation with WPW syndrome and treated the patient by DC shock (200 J biphasic desynchronize) successfully. After DC shock, the patient was stable with a sinus rhythm of 70 bpm and blood pressure of 120/90 mmHg; the ECG shows a sinus rhythm heart rate of 70 bpm, delta waves, wider QRS, and short PR suggesting WPW syndrome with the right side accessory pathway (Figure 1(b)). The more precise and detailed echocardiogram was done by an experience echographer in our hospital showing situs solitus, levocardia, left side aortic, and Ebstein types B-C; the area of the right atrium is 37 cm^2^ (Figure 2(a)), ASD diameter is 8.3 mm with left to right shunt (Figure 2(b)), tricuspid regurgitation (mild to intermediate), and left ventricular EF of 50%. According to the consultants of our heart team member including congenital cardiac surgeons, electrophysiologist, and congenital cardiologists, we planned to do EP study first for ablating the accessory pathway predicted location which was on the right side and secondly to do the modified Carpentier technique to repair the tricupid valve and right atrial, and right ventricle. And we used maze procedure to ablate atrial fibrillation and ASD closure in the operation.

**Figure 3:**
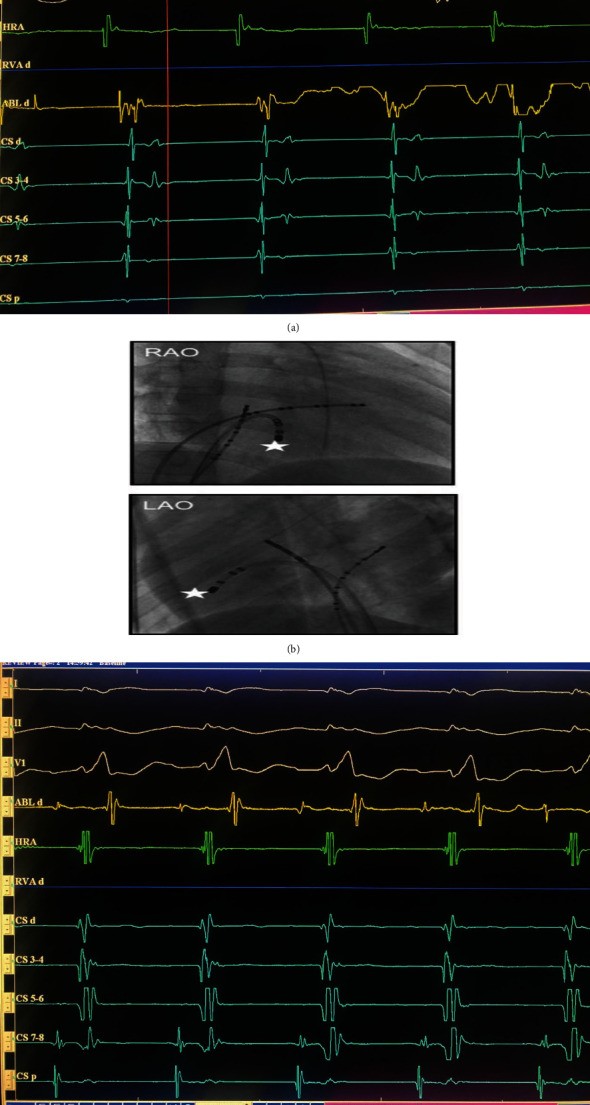
(a) AP potential in EGM in the right lateral of the right atrium. (b) We used the long sheath SL1 to stabilize the ablation catheter to the ablation site and improve the contacting of ablation catheter and myocardium (RAO and LAO views, white star shows target point to ablation). (c) AP potential disappears after ablating the target site.

**Figure 4:**
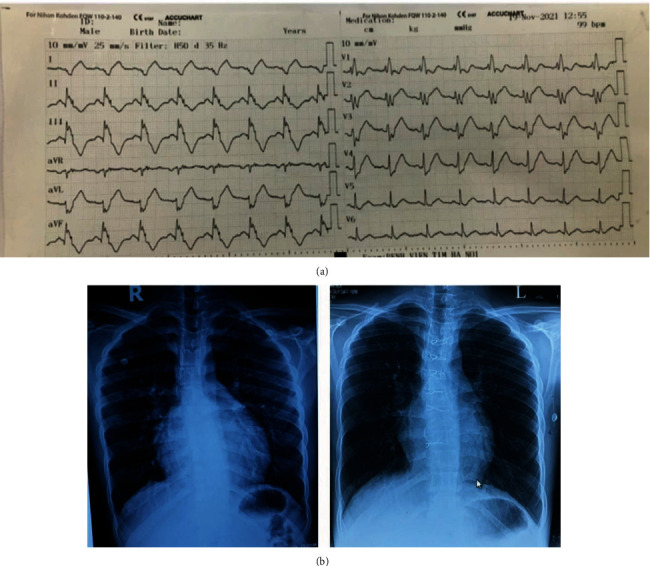
(a) ECG after ablating shows sinus rhythm of 99 bpm and right bundle branch block. (b) Thoracic X-ray shows smaller cardiac-pulmonary index.

In the EP room, we detected the accessory pathway (AP) potential using the intracardiac electrogram in the right lateral of the right atrium (Figure 3(a)). We used the long sheath SL1 to stabilize the ablation catheter to the ablation site and improve the contacting of ablation catheter and myocardium (Figure 3(b)). After ablating in 5 seconds, the AP potential, the delta wave disappeared, and surface ECG shows narrow QRS and long PR interval of 130 ms (Figure 3(c)). The ECG after ablating shows the following: sinus rhythm of 99 bpm and right bundle branch block (Figure 4(a)). We did operation 2 days after with successful repair modified Carpentier technique, Maze procedure, and ASD closure. The patient was stable after that and discharged after 3 days in the ICU and 7 days in the CCU. Thoracic X-ray shows smaller cardiac-pulmonary index (Figure 4(b)). Echocardiogram shows no ASD, normal EF of 50%, and no abnormal shunt.

## 2. Discussion

In reality, the recurrent rate of right lateral pathway is really high; it ranges from 2.9% to 7% [1]. The high recurrent rate is associated to the poor electrode-tissue, instability of the ablation catheter, and the interval time from the onset of the energy application to the loss of AP conduction. The incidence of right lateral and right septal accessory pathway in Ebstein is ranging from 6 to 26% [2, 3]. In this case by comparing ECG before and after ablation, we can realize the present RBBB when the right lateral AP was ablated successfull. This absence of RBBB can be explained by the present of the right side AP [4, 5]. The effectiveness of using long sheath SL 1 to detect AP potential and stabilize the ablation catheter is proven in our case. In Hanoi Heart Hospital, Nguyen et al. report 52 cases of Ebstein anomaly completely repaired by modified Carpentier reconstruction (Cone reconstruction) from 2005 to 2010 with a mortality rate of 1.92% [6]. In this case, the co-operation of EP study department, cardiac surgery department, and congenital heart disease department of our heart team is the key to success of treating this patient. Besides it, by using new technology technique according to the nearest cardiovascular trend in the world, we can improve the outcome, increase the success, decrease the mortality, and the complication rate of our patients.

## Data Availability

The datasets used and/or analysed during the current study are available from the corresponding author on reasonable request.
